# Supplementary material for: Fibration symmetries and cluster synchronization in the Caenorhabditis elegans connectome
Source: PLoS One. 2024 Apr 10;19(4):e0297669. doi: 10.1371/journal.pone.0297669 (PMC11006206; doi:10.1371/journal.pone.0297669)
Supplement: Attachment — Submitted filename: Response_to_Reviewers.pdf [file pone.0297669.s001.pdf]

Thank you for your time and effort in reviewing our manuscript. We appreciate your valuable feedback and suggestions. We hope to have clarified all of the reviewer's observations and implemented their suggestions and we also hope that it now meets the standards of the journal. We honestly believe that these suggestions have aided to improve the quality of our manuscript. We look forward to hearing from you regarding our submission. We would be glad to respond to any further questions and comments that you may have. Please find below the responses to the comments and suggestions related to our submission.

Reviewer #1: This paper discusses application of tools from automorphism groups/symmetries and fibration symmetries to understand synchronization patterns in the locomotion neural sub-networks implicated in the forward and backward gait of the C. Elegans. I found the paper overall quite confusing.

I list below all of my questions/concerns.

Line 50, I could not understand this sentence "Where this synchronization is accomplished when two or more neurons share a set of neurons as their inputs and where these inputs have equivalent connectivity structures; such synchronization is explained further in the paper"

Thank you for your comment. The intention with this line was to briefly explain, without going too much into detail, what are the conditions for two or more nodes to attain synchronicity. I have re-edited this to the following:

The nodes in any of these clusters synchronize due to receiving equal amounts of input from the same or equivalent sets of nodes. This procedure can go on ad infinitum, meaning to be extended for as many existing network nodes. This synchronization is explained in more detail further in the paper.

As now seen in line 50 through 53.

Line 74, what does it mean that biological networks have mild constraints?

Thank you for your comment again. Sorry this was not written appropriately. What we meant to say is:

Finding perfect symmetries in any biological network is highly unlikely, therefore we expect biological networks to exhibit more fibrations symmetries than orbit symmetries, due to the milder constraint of fibers as compared to orbits, (if any symmetries are detected in the first place).

The above sentence now substitutes the previous and can be found in lines 74 to 77.

Line 117, I don't understand the relevance of studying chemical gap junctions and electrical synapses "in isolation". Wouldn't the patterns of synchronization observed in reality depend on both? This is a particularly serious criticism considering that the paper is trying to apply concepts from physics and mathematics to better understand biology

Thank you for this observation, we hope to clarify the issue you have raised by explaining our motivations for taking the steps that we have done in this paper. Our answer seen below has also been inserted into our paper.

It is true that any nervous system relies on both gap junctions and chemical synapses. However, it is known that both types of connections contribute differently to the overall activity of a given circuit. For

example, gap junctions are known to be more important for the transition between the backward and forward circuits rather than the generation/propagation of these behaviors separately [43]. Moreover, specifically in the backward circuit, gap junction removal does not disrupt A type motor neuron synchronicity, but reduces the strength of these motor neurons. This happens because the inter-neuron AVA activates A type motor neurons through acetyl cholinergic signaling and is the major driver of this reverse locomotion [44]. Additionally, as will be seen, simulations of a network of neurons solely composed of gap junction connections with no driving input stimuli will settle into a global synchronous state, meanwhile a network composed solely by chemical synapses and no external stimuli will settle into different clusters fully dependent on the network's symmetries Altogether, despite agreeing that studying these connections in separate might not be ideal, there is previous experimental evidence for their individual contribution.

The above can be now seen in lines 120 through 135.

Line 245, A graph is defined in terms of two sets, the sets of nodes and the sets of edges, but here there are two distinct sets of edges: chemical and electrical, so a proper definition would be G(N,E_gap,E_synapse)

Thank you for this feedback, I find it very useful. My intention is to introduce the reader to the definition of a graph in terms of these two sets without further complications. I will incorporate your input towards the end of this sentence indicating that the set of edges E_G can be further decomposed into two different types of edge sets E_chem, E_gap.

The set of edges E_{G} can be further decomposed into two separate types representing chemical synapses E_{Chem} and gap junctions E_{Gap}, where are a more formal definition of a locomotion circuit as a graph would be: $G=(N_{G}, E_{Chem}, E_{Gap})$.

The above can be now seen in lines 265 through 268. Smaller additional changes have been applied in the lines immediately below as can be noticed by the color blue.

Line 301: Is the minimal graph fibration the same as the minimal balanced coloring? If yes, I would make it explicit

Yes they are and we have made the appropriate indication in our manuscript. Such a statement reads as:

Throughout this paper we refer to the minimal graph fibration as the minimal balanced coloring of a graph.

This line can be found throughout lines 343 to 344.

Line 308 "Nodes in the same fiber synchronize" however both physically and biologically this is only true if the synchronous solution is stable

Yes this is true and we state a similar fact in the description of figure 1. But it is clear that such a piece of information should be contained in the main body of the manuscript. So we have added the following line at the end of this paragraph with some additional modifications as seen now in lines 352 to 359:

Although the natural occurring biological variations from neuron to neuron can be expressed as variations in the same set of parameters among the admissible ODEs and the physical propagation of

signals between neurons can be incorporated through different time retardation terms. All these modifications do not necessarily destroy the expected synchronization of nodes in the same fiber, but do indeed reduce the parameter space of the equilibrium solution where such is attainable [67, 68]. Therefore we forgo integrating these for the sake of simplicity of our models.

67. Dahms T, Lehnert J, Schöll E. Cluster and group synchronization in delay-coupled networks. Physical Review E. 2012;86(1):016202.
68. Cao B, Wang YF, Wang L, Yu YZ, Wang XG. Cluster synchronization in complex network of coupled chaotic circuits: an experimental study. Frontiers of Physics. 2018;13:1–11.

Line 311: "it is possible for two or more fibers to synchronize" -- this should not be possible unless the synchronous solution is an equilibrium

Thank you for your input. I believe we are on the same page, we have expanded on this through the answering of your previous comment. Additionally we now state the following:

Also based on theory, there are no restrictions on the differences in behavior between two nodes in two different fibers, and it is possible for two or more fibers to synchronize [27], such is dictated by the set of equilibrium solutions of an ODE system.

As seen through lines 346 to 349.

Line 314 "We find the fiber partitioning algorithmically by initially coloring all nodes and arrows with a unique color. After that, the algorithm recolors all nodes with the same number of colored inputs with a new color, including their outgoing arrows. These procedures continue until no recoloring is possible." -- this sounds very much like the algorithm proposed in "Chaos
. 2011 Mar;21(1):016106. doi: 10.1063/1.3563581." This algorithm produces the minimum balanced coloring very efficiently.

In the following two lines after this one we stated that: "We apply this method through the algorithm proposed by Kamei & Cock [67] and implement it through the code developed in [38]."
In Kamei & Cock it states that "The algorithm we use is a generalization of that of Belykh and Hasler [9] to consider multiple arrow types, or equivalently a simplification of the Aldis [4] algorithm, where phase one is eliminated at the cost of counting absent arrow type/tail-node color combinations."

To avoid any citations issues we rewrote the previous two lines as:

This is the algorithm developed by Kamei & Cock [69], which is a generalization of the algorithms developed by Belykh and Hasler [70] and by Aldis [66] and implemented through the recreated code in [38].

This can now be found in lines 363 to 365.

Section Building blocks -- I found this section very difficult to follow. I encourage the authors to rewrite this section by making sure that it is accessible to non-experts

We thank the reviewer for this critical observation and agree that this section needed some more additional work and refinement in our explanations. We have modified two different sections to fix this

issue, first we fixed a previous section which is a precursor to the building blocks section. Here we caught some errors and added additional definitions; for this please go to the subsection titled "Fibration, input trees and the minimal balanced coloring" found through lines 296 to 331.
The building block section has been worked on and the ordering of its elements has been re-organized. Please find this through lines 442 to 554.

Eq. (9) The external current term effectively modifies the symmetries and fibrations of the networks, unless currents are always picked to be the same for nodes in the same orbit/fiber. This should be discussed

Thank you for your comment, we will like to point out that we did make the reader aware of our decision in lines 571 to 574 just below Eq. 9 (now Eq. 10) as seen:

The left and right versions of an inter-neuron pair, which govern the activation of the downstream motor-neurons DA, DB, VA, and VB responsible for activating wall muscles that produce locomotion, are mainly selected to receive the external stimuli through Iexti [52].

After further introducing the networks we did make the reader aware of this again in lines 872 through 875 in the Network Simulation section as seen below.

To achieve this, we drive the four networks by delivering the same stimulus through left-right inter-neuron pairs. These left-right inter-neuron pairs belong to the same partition in all four networks, therefore not breaking the fiber partitioning.

But to introduce further clarification we added the following additional lines after lines 576 - 578, two paragraphs below Eq.(9) (now Eq. 10):

Neurons that are considered to be synchronous through their graph analysis (like inter-neuron pairs) must receive the same input or else these will not attain synchronicity.

Eq. (16) makes it clear that the external current includes a noisy term. I think this deserves very careful consideration and discussion within the manuscript. This noisy term should act differently from the other terms in Eq. (16), since in practice noise will never be the same at different neurons. It should be clarified what is the correlation between noise terms at different nodes/neurons.

We thank the reviewer for their comment. We have decided to add the following lines below Eq. 16 (now Eq. 17):

A consensus agreed upon depiction of dynamics of neurons would have independent noise for each neuron. If this case were applied, neurons belonging to the same fiber will indeed no longer have an exact synchronization V_{i}(t) = V_{j}(t). Depending if the strength (amplitude) of the noise term is kept small relative to the constant driving stimuli or the epsilon term in the LoS measure (measure introduced in the following section), the latter will give a result close to 1 indicating near synchronicity. As it will be seen, in our models inter-neuron pairs are the ones to receive external stimuli and therefore drive the network. If each inter-neuron receives independent noise, the synchronicity of all other neurons will not be affected by this implementation. This is due to the fact that all of the networks are left-right symmetric giving way to neurons in each fiber receiving both signals provenient from the driven inter-neuron pair. By such there are no motor-neurons that only receive a signal from only one of the driven inter-neurons which would lead to a breaking of the expected fiber partitionings.

This new addition can be found throughout lines 596 to 608.

Line 541: "Noise dynamics were implemented through a modified stochastic Runge-Kutta method as proposed in [87], where the update time step for the noise is dt1/2." I found this discussion generic. I think it would be better to add a detailed description of the method in an appendix/supplement

Thank you for your feedback, I believe that PLOS One standards have it such that the supplementary section is to be used for essential supporting files and multimedia files. Due to this I have left this section in the manuscript.

Done.

Line 562: "To apply the LoS metric to our simulations, we allow each network to reach a stable state after the initial transients by running it for a sufficient amount of time." -- what if the network does not reach a stable state after the initial transient? By stable state do the authors mean an equilibrium?

Thank you for your comment and for identifying this lack of a definition, yes, this is exactly what we meant. A stable state is meant to be the same as an equilibrium solution. We have added the line below right after line 562-563 (now line 680-681):

Attaining such is guaranteed due to the equilibrium solutions of our ODEs being attractors as will be seen later in the paper. (Note: we use the term stable state equivalently to that of an equilibrium solution of an ODE system throughout this paper).

Additionally the equilibrium solutions are shown to be attractors in stability analysis done on our ODE models within the *simulation test 2* section.

Line 591 I found this confusing "The resulting partitionings and their cluster cells are the same for both binary and integer weighted networks since the graph structure G is the same regardless of edge type. However, the input tree for a fiber associated with a specific neuron is likely different between binary and weighted versions." I thought weights would play a role? If that's not the case, why is that?

Thank you for your comment and we hope to clarify any confusion in this. We have constructed the weighted versions of binary networks as stated in lines 706 and 711 as:

In addition we also work with the integer edge weighted version of the aforementioned networks that respect the symmetrization done in [37] and their fiber partitionings. For the latter the weight of an edge between two fibers was taken to be the rounded-up integer of the average of all the edges present in the original connectome of [8] between the cluster cell of the fibers under consideration.

Through this construction we are guaranteeing the partitioning (clustering) of the nodes in any of these weighted networks remains the same as the ones in the binary case.

Take as an example that in the original connectome (Varshney) we have the following edges between the two fibers composed by {[AVAL, AVAR], [VA08, VA09]} present in the backward locomotion sub-network:

AVAL → VA08 Weight: 9

AVAL → VA09 Weight: 7

AVAR → VA08 Weight: 12
AVAR → VA09 Weight: 6

The average weight of the edges from the fiber composed by the interneurons to those composed by the motor-neurons is 8.5 which rounding up provides us with a weight of 9. In the constructed weighted version we will have all of the edges from the fiber composed by the interneurons to those composed by the motor-neurons have a weight of 9.

Line 629 Obit-> Orbit

Thank you for this grammatical correction, the change has been implemented.

I found the sentences starting at lines 689 and 692 to contrast each other. How are the initial voltages set?

Thank you for catching this. Indeed these lines did not explain correctly our implementation. We have modified the first sentence to remove any contrast. The first sentence now read as:

We set the initial voltages to be normally distributed around resting state potential of -37mV and…

Please find this in lines 821 and 822.

Same paragraph -- what is the standard deviation of the synaptic variable? Is this the noise term?

Thank you for your comment. We defined such in (now) lines 824 to 826 and it varies from 0 to 0.1. The synaptic variable is not a noise term but is part of the chemical synapses of one of our ODE models as it can be seen in (now) Eq 15. Moreover, the synaptic activity variable represents the level of activity at a synapse, and its dynamics are influenced by the membrane voltage of the neuron and the parameters that define the rise and decay of synaptic activity. The equilibrium value of this variable is determined by a combination of the neuron's membrane voltage and the parameters of the sigmoid function that models the synaptic activity's response to changes in voltage.

I have added a paraphrased version of the sentence above in lines 629 to 631, it reads:

The synaptic activity variable depicts the activity magnitude at a synaptic junction, and its behavior is affected by the neuron's membrane potential as well as the parameters determining the increase and decrease of synaptic activity.

Line 700: syntactic variable?

Thank you for finding this grammatical error, the appropriate change has been made.

Line 723: "we drive the four networks
by delivering the same stimulus through left-right inter-neuron pairs" what is the relevance of this? Are left-right inter-neuron pairs always in the same orbit or same fiber? The authors should distinguish perturbations that drive the system away from the synchronous solution, from those that do not.

Thank you for your input. I believe by answering the reviewer's comments on Eq. 9 we have partially answered this question, indicating that inter-neuron pairs are always in their own unique fiber.

Regarding the part that has not been answered, we appreciate your insight in helping improve this paper. We have decided to add the following at the beginning of the next paragraph from line 723 (now line 872) clarify such:

The external stimuli in any of these networks ultimately changes the equilibrium solution of its respective ODE due the nature of these equations. The sinusoidal and noise stimuli will lead the voltages of the neurons away and towards equilibrium periodically and aperiodically respectively. By such these two stimuli can be regarded as perturbations if kept relatively small compared to the constant term stimuli of Eq 16 which dictates the equilibrium point solutions. Additionally, it is important to ensure that the external stimuli do not induce any instabilities that could complicate the interpretation of results such as bifurcations or chaos which a fiber analysis of a graph will not be able to predict.

Find this new addition between lines 876 to 884.

Line 740: "ensure that the external stimuli do not induce any instabilities that could complicate the interpretation of results" -- what does this mean and how does this lead to the Jacobian analysis?

We apologize for any confusions and hope to clarify our intentions, thanking the reviewer for pointing the issue out. What was meant by *instabilities* in this sentence is any deviations from the expected equilibrium solutions that are in accordance with the expected fiber synchronicities, such as bifurcations. The Jacobian analysis serves as a tool to determine the input current space at which these bifurcations do not appear and by staying below these values at which these bifurcations occur, we ensure no further complication for our analysis.

Line750: "As a rule of thumb, if all eigenvalues are real and negative, then the system is stable, meaning that any small perturbations from the equilibrium point will dampen over time, and the system will return to its steady state." That is not a rule of thumb. Also does this mean we are studying stability of fixed points? What happens when the external current is sinusoidal? What about the noise term?

Thank you for pointing out the phrasing. You are correct; the stability criterion based on the real parts of the eigenvalues is a well-established mathematical principle, not just a rule of thumb. We used the term colloquially to mean a general guiding principle, but we recognize that it might not be the most appropriate choice of words in this context. We revised the statement to more accurately reflect the mathematical rigor behind the principle by substituting it with:

According to the established principles of linear stability analysis, if all eigenvalues are real and negative, then the system is stable…

Found in lines 893 to 894.

Concerning the effects of external current and noise, the paper mentions that the gap junction model is stable under any external stimuli as it does not depend on the external current I_ext (line 1073). For Chem networks, when the strength of external stimuli is greater than specific values, a voltage bifurcation arises for the pair of neurons being driven by a constant input, breaking the predicted synchronization from that of the minimal balanced coloring (line 1081).
With this we limit the amplitude of the sinusoidal signal plus that of the noise term to be well below the values at which a constant input induces these bifurcations as seen in table 1. Specifically we keep

the max amplitude of the sum between the constant term, and the sinusoidal or noise term to be at most 95% of the necessary amplitude which would induce an instability (bifurcation). This explanation was given between lines 940 and 943 of the updated manuscript.

Knowing the maximum strength of the external stimuli at which no instabilities appear, we stimulate their respective network + ODE model with a constant input equal to 90% of the external stimuli necessary to cause instability in the network plus a sinusoidal (or noise) stimulus with an amplitude 5% of the instability strength.

To study the stability of the equilibrium of these systems exclusively under one of these two types of inputs (sinusoidal or noise) we would need to implement the method of averaging or the concept of "mean-square stability" respectively. The basic idea is to average these systems over one/a period of time of external stimuli. If the max amplitude is capped, as we have done, their effect will average out to zero, leading the dynamics to sway around the stability point which would be that of the network with no input.

Additionally, I didn't understand the relevance/differences between the three simulation tests proposed. This point requires in my opinion careful consideration and a clearer discussion.

We apologize if this was not too clear and hope to clarify our intentions. To put it simply, we had the aim to show the reader how the networks behave under different conditions and how different fibers synchronize with each other, where this was attempted to be shown contrasting simulation tests 1 and 2. Simulation test 3 has the intention to inspect what happens to the expected fiber synchronization if the structure of the network remains the same but its edges take on everso more random values (increasing the variance around the original weight of 1 of these edges) while being driven by an external stimuli with an amplitude no greater than 0.61pA, below any of the values that would induce instabilities in any of our networks as seen in Table 2.

To capture the above in our paper we have added the sentences below in lines 807 through 819:

In this section, we perform 3 types of numerical simulations according to the specified ODEs in the Admissible ordinary differential equations section and compare their outcomes to those predicted by orbits and fibers partitionings. Throughout these simulations we aim to showcase how the networks behave under different stimuli conditions and how different fibers synchronize with each other depending on this by contrasting the outcomes of simulation test 1 and 2. Within test 2 we seek to also determine the ranges in which the partitioning predictions are valid as external stimuli, if large enough, can induce instabilities. After such we explain how we stimulate these networks while remaining within the regime to not induce these instabilities. Simulation test 3 has the intention to inspect what happens to the expected fiber synchronization if the structure of the network (with external stimuli) remains the same but its edges take on evermore random values, we find that some version of synchronicity can still be captured.

With additional clarifications in between lines 984 to 988.

In this simulation, the driving signals are composed of a constant input of 0.1pA plus an oscillatory term with a frequency of 2Hz for inter-neurons and 1Hz for motor-neurons with an amplitude set to 0.5pA plus a Gaussian random walk scaled between −0.01pA and 0.01pA. This gives a max amplitude of 0.61pA, below any of the values that would induce instabilities in any of our networks as seen in Table 2.

There are smaller changes between these two examples above that have been highlighted in blue.

Additionals: To finish this document we would like to address that the link to our data and software was changed during the reviewing process and therefore the link provided did not work. This was an oversight from our part and apologies for the inconvenience this has caused. We have now corrected the link in the paper and find that it is working. We would like to end this document by once again thanking the reviewer for their comments and suggestions hoping to have addressed them all in a way that satisfies the reviewer and the editors of the journal.
